# One-year worsening heart failure and myocardial T1 mapping in patients with wild-type transthyretin amyloid cardiomyopathy undergoing tafamidis treatment

**DOI:** 10.1016/j.ijcha.2026.101934

**Published:** 2026-04-24

**Authors:** Toshiro Kitagawa, Kotaro Hamamoto, Daiki Okamoto, Yuki Ikegami, Yoshiharu Sada, Fuminari Tatsugami, Yukiko Nakano

**Affiliations:** a Department of Cardiovascular Medicine, Hiroshima University Graduate School of Biomedical and Health Sciences, Hiroshima, Japan; b Department of Diagnostic Radiology, Hiroshima University Graduate School of Biomedical and Health Sciences, Hiroshima, Japan

**Keywords:** Heart failure, Tafamidis, Transthyretin amyloid cardiomyopathy, T1 mapping

## Abstract

**Background:**

There is a paucity of data regarding short-term outcomes and treatment responsiveness of myocardial T1 mapping via cardiac magnetic resonance (CMR) in patients with transthyretin amyloid cardiomyopathy (ATTR-CM) undergoing tafamidis therapy.

**Methods:**

We retrospectively studied 60 wild-type ATTR-CM patients who underwent baseline CMR to measure native myocardial T1 value (T1_native_) and extracellular volume fraction (ECV), followed by tafamidis treatment. Cardiac biomarkers, including high-sensitivity cardiac troponin T and N-terminal pro-B-type natriuretic peptide (NT-proBNP), were measured at baseline. We followed a one-year composite of worsening heart failure (WHF; hospitalization and/or intensification of diuretic therapy for heart failure). Additionally, 51 patients underwent follow-up CMR and measurements of cardiac biomarkers one-year after the initiation of tafamidis treatment.

**Results:**

Patients with WHF (n = 12) exhibited significantly elevated baseline T1_native_ and ECV than those without WHF, and their optimal cutoffs in predicting WHF were 1447 ms and 48.7 %, respectively. Multivariate analysis adjusted for Mayo or National Amyloidosis Center stages identified T1_native_ of ≥ 1447 ms as an independent predictor of WHF, with hazard ratios of 15.2 and 9.3, respectively. A notable proportion of patients exhibited a reduction in T1_native_ (39 %) and ECV (47 %) after one year of tafamidis treatment. Post-treatment changes in T1_native_ were correlated positively with changes in NT-proBNP concentration (r = 0.40, *p* = 0.0036).

**Conclusions:**

In wild-type ATTR-CM patients receiving tafamidis, elevated baseline T1 mapping parameters were associated with one-year WHF. T1 mapping parameters, particularly T1_native_, may offer imaging-based evidence of alterations in myocardial characteristics induced by tafamidis.

## 1 Introduction

Tafamidis, a novel disease-modifying treatment for transthyretin amyloid cardiomyopathy (ATTR-CM), functions by inhibiting the dissociation of transthyretin tetramers and subsequent amyloidogenesis. It has been reported to reduce all-cause mortality, incidence of cardiovascular-related hospitalizations, and decline in functional capacity and quality of life [1]. In Japan, tafamidis received approval for the treatment of ATTR-CM in March 2019 [2]. The introduction of tafamidis has heightened clinical interest in the diagnosis and assessment of ATTR-CM.

The Japanese Circulation Society guidelines for cardiac amyloidosis (CA) propose an algorithm for diagnosis using technetium-99 m pyrophosphate scintigraphy [3]. Concurrently, the recommendations for cardiac magnetic resonance (CMR) in CA include the evaluation of cardiac morphology and function through cine imaging and differentiation from other cardiomyopathies using late gadolinium enhancement, T1 mapping, and T2 mapping techniques. Notably, the native myocardial T1 value (T1_native_) facilitates the measurement of the intrinsic signal from the myocardium, and T1 values obtained before and after gadolinium-based contrast administration can be used to calculate the myocardial extracellular volume fraction (ECV). These T1 mapping parameters have been correlated with the disease burden and have demonstrated good diagnostic accuracy [4], [5], [6].

Since the introduction of tafamidis for the treatment of ATTR-CM, there is a need for methods to predict clinical outcomes and monitor changes in myocardial characteristics following treatment initiation. Myocardial T1 mapping may serve these purposes and potentially aid in identifying treatment responders. Nevertheless, data on the short-term prognostic value of myocardial T1 mapping parameters and their early responsiveness to treatment in ATTR-CM patients receiving tafamidis remain scarce. Previous studies have indicated that one-year changes in T1_native_ and ECV following the initiation of tafamidis treatment were not statistically significant [7], [8]. We previously reported preliminary data showing that changes in T1_native_ and ECV after one year of tafamidis treatment were negatively correlated with their baseline values, and the change in T1_native_ was positively correlated with the post-treatment change in serum N-terminal pro-B-type natriuretic peptide (NT-proBNP) concentration [9]. However, to effectively employ T1 mapping techniques in managing ATTR-CM under tafamidis treatment, it is crucial to further explore how changes in post-treatment myocardial T1 mapping parameters correlate with other clinical parameters and outcomes.

In this study, we assessed the prognostic significance of myocardial T1 mapping parameters in predicting the progression of heart failure over a one-year period in patients with ATTR-CM undergoing tafamidis treatment. Additionally, we aimed to elucidate the one-year responsiveness of myocardial T1 mapping parameters to tafamidis treatment, in comparison with other myocardial parameters, cardiac biomarkers, and clinical outcomes.

## 2 Methods

### 2.1 Study participants

This study represents a post hoc analysis of our prior research [9]. Between April 2021 and September 2024, we retrospectively enrolled 75 consecutive patients diagnosed with ATTR-CM who underwent CMR at Hiroshima University Hospital. In accordance with the Japanese Circulation Society guidelines for diagnosing CA [3], the definitive diagnosis of ATTR-CM necessitates histological confirmation of amyloid deposition in the cardiac or extracardiac tissue. Consequently, the diagnosis of ATTR-CM was established through histochemical detection of amyloid and transthyretin deposition in the myocardium or extracardiac tissue with 99 mTc-labeled pyrophosphate uptakes in the myocardium. Wild-type ATTR-CM was identified by the absence of mutations in transthyretin, as determined by genetic testing. Exclusion criteria included the presence of other specific etiologies that influenced myocardial T1 values (e.g., myocardial infarction, hemochromatosis, and Fabry disease). Patients with severe anemia (hemoglobin concentration < 10 g/dL) were also excluded from the study. Individuals with advanced chronic kidney disease (estimated glomerular filtration rate < 30 mL/min/1.73 m^2^ or those undergoing dialysis) or a history of allergy to gadolinium-based contrast agents did not undergo post-contrast imaging, nor did those who declined contrast administration. Patient characteristics, including pre-imaging serum concentrations of cardiac biomarkers (serum high-sensitivity cardiac troponin T [hs-cTnT] and NT-proBNP) measured within one month prior to the baseline CMR scan, were extracted from medical records. All patients were clinically stable and exhibited no signs of congestive heart failure at the time of imaging and blood testing (New York Heart Association class I or II). All patients were categorized based on their cardiac biomarker levels (Mayo staging) [10] or based on NT-proBNP levels and estimated glomerular filtration rate (National Amyloidosis Center [NAC] staging) [11].

The indications for tafamidis administration were determined in accordance with the guidelines for its appropriate use in Japan [2]. Tafamidis (61 mg), which is bioequivalent to 80 mg of tafamidis meglumine [12], was administered within one month after the baseline CMR examination.

This study adhered to the principles outlined in the Declaration of Helsinki. The Ethical Committee for Epidemiology of Hiroshima University approved the study protocol (approval no. E2018-1364). Clinical information was collected and data analysis was conducted retrospectively using medical records. Informed consent was obtained using an opt-out method.

### 2.2 CMR protocol

The CMR protocol, which includes cine CMR and both pre- and post-contrast T1 mapping sequences using an Ingenia 3 T CX scanner (Philips Medical Systems, Best, The Netherlands), has been described previously [9]. Cine images were acquired in the short-axis and three long-axis views employing a cine steady-state free precession sequence to evaluate the left ventricular (LV) morphology, mass, and function. T1 mapping was executed using a 13-heartbeat steady-state free procession, a single-breath-hold modified Look-Locker inversion recovery sequence across five short-axis slices (from base to apex). The parameters for the T1 mapping sequence were as follows: slice thickness, 10 mm; TR/TE, 2.2/1.0 ms; flip angle, 20°; field of view, 300 × 300 mm; sampled matrix size, 160 × 155 mm; SENSE factor, 2; and five images from three inversions (3 + 3 + 5) with a one heartbeat pause preceding the second and third inversions and an adiabatic prepulse. Post-contrast T1 mapping was conducted using identical parameters, 15 min after the administration of gadolinium-BTDO3A (Gadavist; Bayer Schering Pharma, Berlin, Germany) at a dosage of 0.1 mmol/kg.

### 2.3 CMR image analysis

CMR images were independently evaluated by an experienced cardiologist and radiologist (TK and FT) using commercially available software (ShadeQuest/ViewR and ViewC, Yokogawa Medical Solutions Corp., Tokyo, Japan). The left ventricular (LV) parameters on cine images and T1 mapping were determined as described previously [9], [13]. In cine images, the papillary muscles were incorporated as part of LV cavity volume, and endocardial LV borders were manually delineated at end-diastole and end-systole. The LV end-diastolic and end-systolic volumes were calculated using Simpson’s rule, and the LV ejection fraction (LVEF) was determined as (end-diastolic volume − end-systolic volume)/end-diastolic volume. The LV end-diastolic volume was adjusted for body surface area (mL/m2) and expressed as the LV end-diastolic volume index (LVEDVI). LV mass was assessed at end-diastole using five slices from the base to the apex of the LV myocardium and normalized to the body surface area (g/m2), expressed as the LV mass index (LVMI). To determine T1_native_ per patient, a region of interest was delineated to encompass the entire LV myocardium per short-axis slice, carefully excluding the papillary muscle, LV cavity, and epicardium. Myocardial segments that were not clearly identifiable owing to motion artifacts were excluded from the analysis through a consensus between the two readers. The average T1 value was derived from five slices of the base-to-apex LV myocardium in each patient. The normal T1_native_ value at our institution was previously established in 10 subjects with no clinical evidence of LV cardiomyopathy and normal CMR findings (1283 ± 34 ms) [12]. The myocardial ECV was determined using commercially available Ziostation 2 software (Ziosoft Inc., Tokyo, Japan). The pre- and post-contrast myocardial T1 values were measured on five slices from the base to the apex of the LV myocardium, and the average ECV per patient was derived from the 16 American Heart Association myocardial segments. The ECV was calculated as follows: myocardial ECV (%) = (1 − hematocrit) × (ΔR1 myocardium) / ΔR1 blood) × 100. Hematocrit data were collected on the same day as the CMR scan.

### 2.4 Follow-up protocol

Within the entire cohort, we monitored the incidence of a composite measure of worsening heart failure (WHF) over a one-year period following the baseline CMR scan. To sensitively detect short-term exacerbations of heart failure, WHF was defined as hospitalization and/or intensification of diuretic therapy (specifically, an increase in the dosage of loop diuretics) in response to signs and symptoms of decompensated heart failure. Follow-up data were collected through a review of hospital records, telephone interviews with patients, and communication with primary care physicians. All endpoints were determined by a consensus between the two reviewers.

A subset of the entire cohort who underwent both baseline and follow-up CMR imaging was retrospectively enrolled to investigate the treatment responsiveness of myocardial T1 mapping parameters. The follow-up CMR examination was conducted in a manner consistent with the baseline assessment, occurring one year (between 12 and 13 months) after the initiation of tafamidis treatment. Furthermore, the serum concentrations of hs-cTnT and NT-proBNP were measured at the time of the follow-up CMR scan. The endpoints of the study were defined as changes in myocardial T1 mapping parameters and serum biomarkers, reported as ΔT1_native_, ΔECV, Δhs-cTnT, and ΔNT-proBNP, representing the differences between follow-up and baseline values (the follow-up minus baseline values).

### 2.5 Statistical analysis

Serum hs-cTnT and NT-proBNP concentrations are expressed as medians with interquartile range; other continuous variables are expressed as means with standard deviation. Student’s *t* test or the Mann − Whitney *U* test was used to compare continuous variables between groups. Categorical variables are reported as number or proportion (%) and were compared with Pearson’s chi-squared test. T1_native_ and ECV were tested with a receiver operating characteristic curve to determine the optimal cutoff values for predicting WHF. The rates of WHF were estimated with Kaplan–Meier curves and compared with a log-rank test. A Cox proportional hazard regression model was used to assess predictors of WHF. The paired *t*-test was used to compare baseline and follow-up T1_native_ and ECV values. Potential correlations among ΔT1_native_, ΔECV, their baseline values, and the changes in cardiac biomarkers were assessed using Pearson’s method. Interobserver reproducibility of T1_native_ and ECV was determined by calculating the 95 % confidence interval (CI) for mean difference and 95 % limits of agreement. P < 0.05 was considered significant. Analyses were performed using JMP Pro 17 statistical software (SAS Institute, Cary, NC, USA).

## 3 Results

### 3.1 Baseline characteristics of the participants

**Fig. 1:**
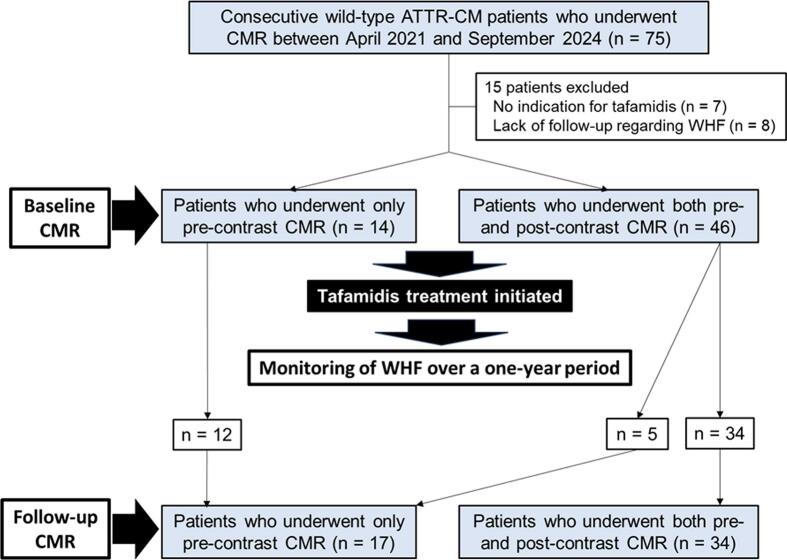
Study flowchart. ATTR-CM − transthyretin amyloid cardiomyopathy; CMR − cardiovascular magnetic resonance; WHF − worsening heart failure.

All 75 patients had wild-type ATTR-CM diagnosed by genetic testing. Patients who were either not indicated for tafamidis (n = 7) or who could not be successfully monitored for the occurrence of a composite of WHF (n = 8) were excluded. Consequently, 60 patients were enrolled in this study. All the 60 patients underwent cine and pre-contrast T1 mapping; 46 of these underwent post-contrast T1 mapping, in which ECV data could be acquired. The CMR follow-up subgroup comprised 51 patients; 34 of these underwent post-contrast T1 mapping, in which follow-up ECV data could be acquired. The flowchart of the study is shown in Fig. 1. The patient characteristics of the entire cohort and the CMR follow-up group are shown in **Table S1**. We observed excellent interobserver agreement for T1_native_, with a mean difference of 2 (95 % CI, 0–5; 95 % limits of agreement, −13-17), and for ECV, with a mean difference of 0.5 (95 % CI, −2.0–1.0; 95 % limits of agreement, −5.3–6.2).

### 3.2 Worsening heart failure after the initiation of tafamidis treatment

Throughout the one-year follow-up period, no patients succumbed, and 12 (20 %) experienced WHF. Of these, five patients required hospitalization due to exacerbation of heart failure and subsequently received intensified diuretic therapy. The remaining seven patients received intensified diuretic therapy during ambulatory care visits. Table 1 shows the comparison of baseline characteristics between patients with WHF and those without. Patients with WHF were predisposed to exhibit elevated baseline levels of cardiac biomarkers and were more likely to be categorized into higher Mayo staging classifications. No significant differences were observed in NAC staging classifications, LVEF, LVEDVI, and LVMI between the groups. Patients with WHF had significantly higher T1_native_ than those without (1458 ± 42 vs. 1415 ± 53 ms; p = 0.014). In patients who underwent baseline post-contrast T1 mapping, the WHF group (n = 8) exhibited a significantly higher ECV compared to the non-WHF group (n = 38) (56.3 ± 6.3 vs. 49.1 ± 8.3 %; p = 0.027).

**Table 1:** Baseline characteristics of the entire cohort according to the presence of WHF.

	WHF presence	WHF absence	p value
	(n = 12)	(n = 48)	
Age (years)	77 ± 6	78 ± 5	0.4
Male sex	11 (92)	42 (88)	0.69
BMI (kg/m^2^)	22 ± 2	23 ± 3	0.77
Heart failure hospitalization within one month	2 (17)	16 (33)	0.26
Hypertension	3 (25)	13 (27)	0.88
Dyslipidemia	2 (17)	11 (23)	0.64
Diabetes mellitus	3 (25)	20 (42)	0.29
Current smoking	1 (8)	7 (15)	0.57
History of atrial fibrillation	3 (25)	16 (33)	0.58
Medications			
Beta-blocker	3 (25)	19 (40)	0.35
ACE inhibitor or ARB	4 (33)	22 (46)	0.43
MRA	7 (58)	21 (44)	0.37
Diuretics	8 (67)	38 (79)	0.36
SGLT2 inhibitor	2 (17)	15 (31)	0.32
Blood testing			
Hemoglobin (g/dL)	13.6 ± 1.6	13.6 ± 1.7	0.99
eGFR (mL/min/1.73 m^2^)	51.1 ± 17.3	49.9 ± 15.6	0.82
hs-cTnT (ng/mL)	0.069 (0.056 − 0.12)	0.056 (0.043 − 0.081)	0.067
NT-proBNP (pg/mL)	3513 (1794 − 4662)	1871 (1091 − 2965)	0.041
Mayo stages			0.038
I	1 (8)	17 (35)	
II	4 (33)	20 (42)	
III	7 (58)	11 (23)	
NAC stages			0.11
I	4 (33)	27 (56)	
II	4 (33)	16 (33)	
III	4 (33)	5 (10)	
LV parameters			
LVEF (%)	50.7 ± 8.3	55.3 ± 11.9	0.21
LVEDVI (ml/m^2^)	82.9 ± 15.9	74.5 ± 16.8	0.12
LVMI (g/m^2^)	102.4 ± 29.3	91.1 ± 22.3	0.15
T1_native_ (ms)	1458 ± 42	1415 ± 53	0.014
ECV (%)	56.3 ± 6.3 (n = 8)	49.1 ± 8.3 (n = 38)	0.027

Serum hs-cTnT and NT-proBNP concentrations are expressed as medians with interquartile range. Other data are expressed as means ± standard deviation or numbers with percentage. ACE, angiotensin converting enzyme; ARB, angiotensin II receptor blocker; BMI, body mass index; ECV, extracellular volume fraction; eGFR, estimated glomerular filtration rate; hs-cTnT, high-sensitivity cardiac troponin T; LV, left ventricular; LVEDVI, LV end-diastolic volume index; LVEF, LV ejection fraction; LVMI, LV mass index; MRA, mineralocorticoid receptor antagonist; NAC, the National Amyloidosis Center; NT-proBNP, N-terminal pro-brain natriuretic peptide; SGLT2, sodium glucose cotransporter 2; T1_native_, native myocardial T1 value; WHF, worsening heart failure.

### 3.3 Predictive value for WHF of myocardial T1 mapping

**Fig. 2:**
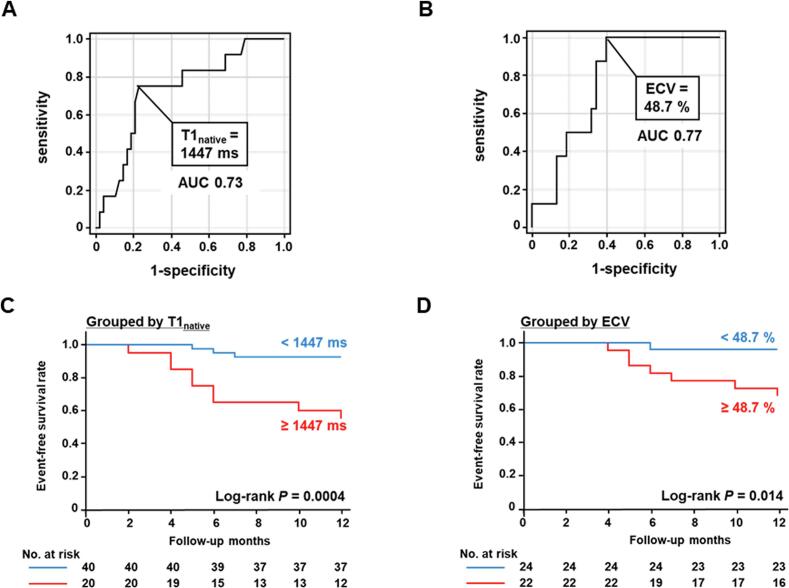
Optimal cutoff values in predicting WHF and Kaplan–Meier curves for WHF. (A) The receiver operating characteristic curve of T1_native_ for predicting WHF. (B) The receiver operating characteristic curve of ECV for predicting WHF. (C) Kaplan–Meier curve for WHF stratified by the optimal cutoff value of T1_native_. (D) Kaplan–Meier curve for WHF stratified by the optimal cutoff value of ECV. AUC − area under the curve; ECV − extracellular volume fraction; T1_native_ − native myocardial T1 value; WHF − worsening heart failure.

**Fig. 3:**
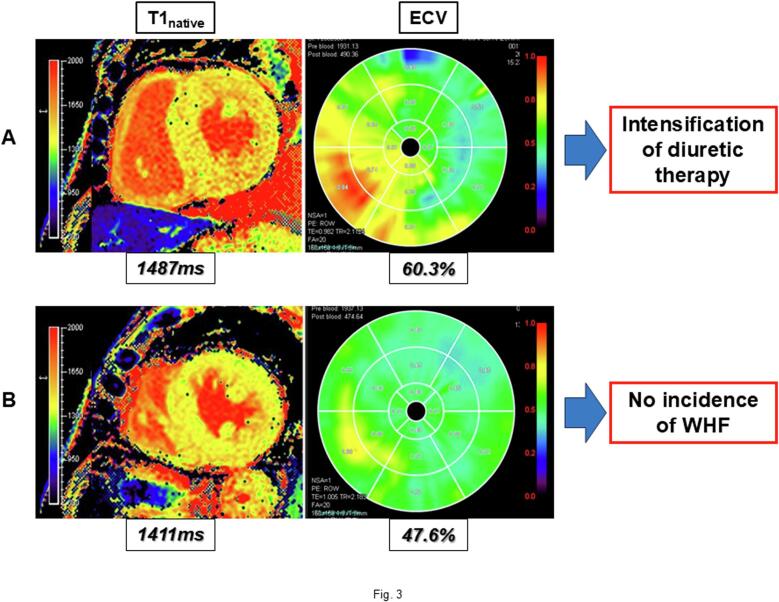
Representative ATTR-CM patients with and without WHF. (A) This patient was an 80-year-old male, and classified as Mayo stage II at baseline. The baseline T1_native_ and ECV were 1487 ms and 60.3 %, respectively. This patient received intensified diuretic therapy (increase in loop diuretic dose) due to symptoms of decompensated heart failure 10 months after the initiation of tafamidis treatment. (B) This patient was a 73-year-old male, and classified as Mayo stage III at baseline. The baseline T1_native_ and ECV were 1411 ms and 47.6 %, respectively. This patient experienced no WHF over the one-year period after the initiation of tafamidis treatment. ATTR-CM − transthyretin amyloid cardiomyopathy; ECV − extracellular volume fraction; T1_native_ − native myocardial T1 value; WHF − worsening heart failure.

The optimal cutoff values of T1_native_ and ECV in predicting WHF were determined to be 1447 ms (sensitivity, 75.0 %; specificity, 77.1 %; positive predictive value, 45.0 %; negative predictive value, 92.5 %; and accuracy, 76.7 %) and 48.7 % (sensitivity, 100 %; specificity, 60.5 %; positive predictive value, 34.8 %; negative predictive value, 100 %; and accuracy, 67.4 %), respectively (Fig. 2**A, B**). Kaplan–Meier curves showed that patients with T1_native_ of ≥ 1447 ms and ECV of ≥ 48.7 % had a higher risk of WHF than those with lower values (Fig. 2**C, D**). **Table S2** shows the comparison of baseline characteristics among patients categorized based on the optimal cutoff values of T1_native_ and ECV. Patients with T1_native_ of ≥ 1447 ms or ECV of ≥ 48.7 % were predisposed to exhibit higher hs-cTnT concentrations, LVEDVI and LVMI, whereas no significant differences were observed in Mayo and NAC staging classifications between the groups. Univariate Cox proportional hazard analyses identified Mayo stage III, NAC stage III, T1_native_ ≥ 1447 ms, and ECV ≥ 48.7 % as predictors of WHF (Table 2). In multivariate Cox proportional analyses adjusted for age, sex, and the Mayo staging classifications, T1_native_ of ≥ 1447 ms remained an independent predictor of WHF in all the 60 patients (hazard ratio, 15.2; 95 % CI, 3.0–76.9; p = 0.0010), whereas ECV of ≥ 48.7 % did not serve as an independent predictor in the subset of 46 patients with ECV data (hazard ratio, 5.4; 95 % CI, 0.6–46.3; p = 0.13) (Table 3). Similarly, when adjusted for age, sex, and the NAC staging classifications, T1_native_ of ≥ 1447 ms remained an independent predictor of WHF in all the 60 patients (hazard ratio, 9.3; 95 % CI, 2,2–40.2; p = 0.0028), whereas ECV of ≥ 48.7 % did not serve as an independent predictor in the 46 patients with ECV data (hazard ratio, 5.7; 95 % CI, 0.7–49.6; p = 0.11) (Table 3). Fig. 3 presents a comparative analysis of the representative patients with and without WHF.

**Table 2:** Univariate analysis of Mayo and NAC staging classifications and T1 mapping parameters in predicting WHF.

	Hazard ratio	95 % confidence interval	p value
Mayo stage III (vs. stage I) (n = 60)	8.6	1.1–70.1	0.044
NAC stage III (vs. stage I) (n = 60)	5.1	1.3–20.3	0.022
T1_native_ ≥ 1447 ms (n = 60)	7.4	2.0–27.6	0.0026
ECV ≥ 48.7 % (n = 46)	8.7	1.1–70.9	0.043

ECV, extracellular volume fraction; NAC, the National Amyloidosis Center; T1_native_, native myocardial T1 value; WHF, worsening heart failure.

**Table 3:** Multivariate analysis of T1 mapping parameters in predicting WHF.

	Model 1	p value	Model 2	P value	Model 3	p value
T1_native_ ≥ 1447 ms (n = 60)	8.0 (2.1 − 30.3)	0.0021	15.2 (3.0 − 76.9)	0.0010	9.3 (2.2 − 40.2)	0.0028
ECV ≥ 48.7 % (n = 46)	7.6 (0.9 − 61.6)	0.059	5.4 (0.6 − 46.3)	0.13	5.7 (0.7 − 49.6)	0.11

Data are expressed as hazard ratio (95 % confidence interval).

Model 1: adjusted for age, sex.

Model 2: adjusted for age, sex, Mayo staging classifications.

Model 3: adjusted for age, sex, NAC staging classifications.

ECV, extracellular volume fraction; NAC, the National Amyloidosis Center; T1_native_, native myocardial T1 value; WHF, worsening heart failure.

### 3.4 Responsiveness of myocardial T1 mapping to tafamidis treatment

**Fig. 4:**
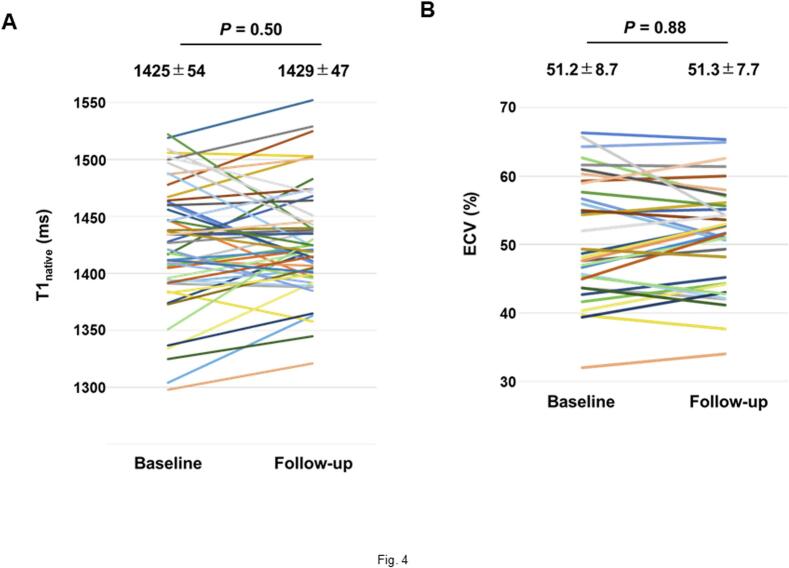
Comparison of myocardial T1 mapping parameters between baseline and follow-up CMR. Both T1_native_ (A) and ECV (B) remained entirely unchanged between baseline and follow-up imaging. Nevertheless, a significant proportion of patients exhibited a reduction in these parameters after one year of tafamidis treatment. CMR − cardiac magnetic resonance; ECV − extracellular volume fraction; T1_native_ − native myocardial T1 value.

**Fig. 5:**
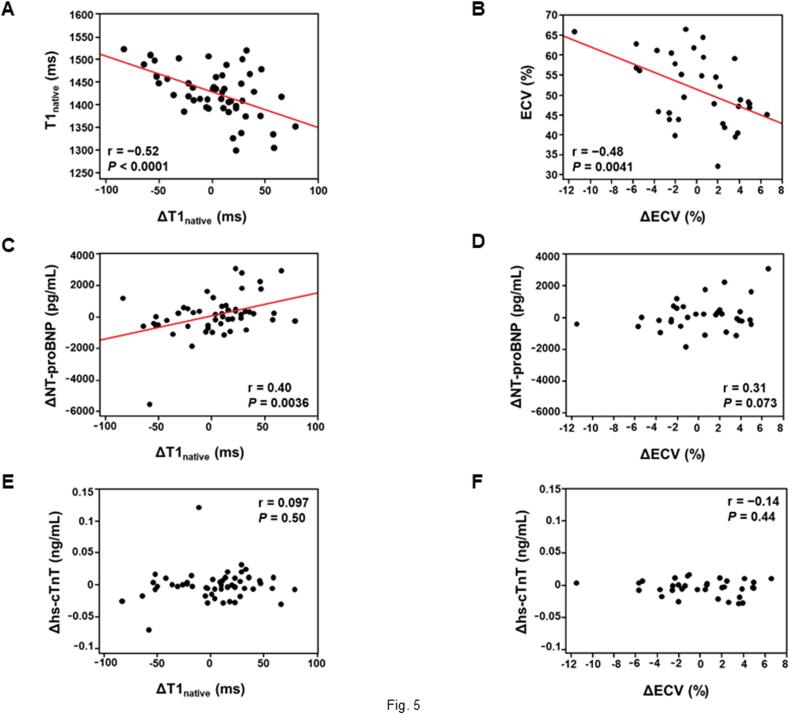
Correlations between changes in myocardial T1 mapping parameters after tafamidis treatment and their baseline values or post-treatment changes in cardiac biomarkers. (A and B) The changes inT1_native_ and ECV exhibited a negative correlation with their respective baseline values. (C-F) The change in T1_native_ exhibited a positive correlation with the post-treatment change in serum NT-proBNP concentration. No other correlations were observed between post-treatment changes in T1 mapping parameters and cardiac biomarkers. ECV − extracellular volume fraction; hs-cTnT − high-sensitivity cardiac troponin T**;** NT-proBNP − N-terminal pro-B-type natriuretic peptide; T1_native_ − native myocardial T1 value.

In the CMR follow-up cohort, there were no significant differences observed in T1_native_ (1425 ± 54 vs. 1429 ± 47 ms; p = 0.50) and ECV (51.2 ± 8.7 vs. 51.3 ± 7.7 %; p = 0.88) between baseline and follow-up imaging. However, a notable proportion of patients exhibited a reduction in T1_native_ (ΔT1_native_ < 0 ms) (39 %, 20/51) and ECV (ΔECV < 0 %) (47 %, 16/34) following the initiation of tafamidis treatment (Fig. 4). There was a moderate correlation between ΔT1_native_ and ΔECV (r = 0.38, p = 0.025). Both ΔT1_native_ and ΔECV were correlated negatively with their baseline values (Fig. 5**A, B**). ΔT1_native_ was correlated positively with ΔNT-proBNP; its correlation with Δhs-cTnT did not reach significance. No correlation was found between ΔECV and changes in cardiac biomarkers (Fig. 5**C–F**). **Fig. S1** shows a representative patient in whom the data of serial CMR scans and cardiac biomarker measurements were acquired before and after tafamidis treatment.

In the comparison between patients with WHF and those without, there were no differences in ΔT1_native_ (4 [–32 – 35 [n = 9] vs. 10 [–19 – 28] [n = 42], p = 0.97) and ΔECV (–1.0 [–3.0 – 3.0] [n = 5] vs. 0.6 [–2.3 – 3.6] % [n = 29], p = 0.73) (**Fig. S2**).

## 4 Discussion

In this study, we assessed the predictive value of myocardial T1 mapping parameters for one-year WHF in patients with wild-type ATTR-CM undergoing tafamidis treatment. Furthermore, we examined the one-year treatment responsiveness of myocardial T1 mapping parameters following the initiation of tafamidis therapy. Our findings are as follows:

1. Elevated T1_native_ and ECV values prior to the commencement of tafamidis treatment were associated with one-year WHF.
2. After adjusting for Mayo or NAC stages, a higher T1_native_ value persisted as an independent predictor of one-year WHF.
3. After one year of tafamidis treatment, a notable proportion of patients exhibited a reduction in T1_native_ and ECV, and changes in T1_native_ and ECV exhibited a negative correlation with their respective baseline values.
4. The alteration in T1_native_ following one year of tafamidis treatment demonstrated a positive correlation with the post-treatment change in serum NT-proBNP concentration. However, no other correlations were observed between post-treatment changes in T1 mapping parameters and cardiac biomarkers.
5. The alterations in T1_native_ and ECV following one year of tafamidis treatment did not exhibit any correlation with one-year WHF.

The potential of T1 mapping parameters to predict short-term exacerbation of heart failure in ATTR-CM patients undergoing tafamidis is noteworthy. Furthermore, this study offers novel insights into alterations in myocardial T1 mapping parameters following tafamidis therapy in relation to their baseline values and subsequent changes in cardiac biomarkers. On the one hand, we observed no correlations between changes in myocardial T1 mapping parameters and the short-term worsening of heart failure. Our results indicate how to utilize CMR and T1 mapping techniques in the clinical practice of ATTR-CM.

Risk stratification in patients with ATTR-CM is essential for effective management and treatment; however, monitoring disease progression in ATTR-CM remains challenging because of the absence of established best-practice guidelines. An expert consensus from Europe has recommended a set of 11 measurable features across three domains for monitoring ATTR-CM progression [14]. These recommendations were formulated prior to the widespread use of tafamidis and did not incorporate the use of CMR. In a prospective study conducted between 2011 and 2015 involving ATTR-CM patients, 95 of 227 (42 %) patients died, with both T1_native_ and ECV serving as predictors of mortality [15]. A previous *meta*-analysis indicated that ECV offers superior prognostic utility for assessing CA compared to late gadolinium enhancement and T1_native_ [6]. A study examining the progression of ECV and its prognostic significance in patients with CA found that changes in ECV were predictive of outcomes [16]. These findings were derived from data on heterogeneous CA patients, including those with light-chain amyloidosis and ATTR-CM, with or without tafamidis treatment. In this study, we demonstrated that baseline high T1_native_ and ECV are associated with a one-year worsening of heart failure in patients with wild-type ATTR-CM following the initiation of tafamidis treatment and that high T1_native_ independently predicts this outcome, irrespective of the clinical stage based on cardiac biomarker levels. To our knowledge, this is the first study demonstrating the utility of T1 mapping in predicting short-term outcomes in patients undergoing disease-modifying treatment for ATTR-CM.

Recent studies have identified the outpatient loop diuretic intensification or initiation as a significant prognostic marker in patients with ATTR-CM [17], [18]. In this study, we defined WHF as hospitalization and/or diuretic intensification resulting from heart failure exacerbations. Our findings indicate that T1_native_ independently enhances the sensitive detection of ATTR-CM patients at an elevated risk of short-term heart failure deterioration. The predictive utility of ECV for one-year WHF did not achieve statistical significance in the multivariate Cox proportional analysis adjusted for the Mayo or NAC staging classifications, potentially because of the limited sample size of ECV. However, unlike ECV, T1_native_ measures a composite tissue signal derived from both cellular and interstitial components, thereby facilitating the assessment of the entire myocardium. We posit that this characteristic, along with its acquisition without the need for gadolinium contrast administration, constitutes a significant advantage of T1_native_ in evaluating the future risk of ATTR-CM.

Consistent with our preliminary analyses, we observed no significant short-term responsiveness of myocardial T1 mapping parameters to tafamidis treatment. Nevertheless, 39 % (T1_native_) or 47 % (ECV) of patients with ATTR-CM exhibited a reduction in T1 mapping parameters following the initiation of tafamidis therapy. The processes of amyloid clearance through endocytosis in fibroblasts and phagocytosis by immune cells have been previously documented [19], [20]. It has been postulated that the equilibrium between amyloid formation and clearance rates may be modified by halting or decelerating amyloid accumulation [21]. The pharmacological effects of tafamidis may lead to a condition in which amyloid clearance from the myocardium slightly surpasses its accumulation. We also identified significant negative correlations between changes in myocardial T1 mapping parameters and their baseline values. Although the underlying cause of these findings remains unclear, the aforementioned mechanism may be more likely to occur when the pre-existing amyloid burden is more pronounced. Notably, we observed a positive correlation between the change in T1_native_ and post-treatment changes in serum NT-proBNP concentration, whereas the correlation between changes in ECV and post-treatment changes in cardiac biomarkers did not reach statistical significance. T1_native_, which represents a composite tissue signal from both myocytes and interstitium, may be effective in detecting meaningful changes in LV overload following tafamidis treatment. The changes in T1_native_ and ECV after one year of tafamidis treatment were not correlated with the one-year progression of heart failure in this study. Therefore, in evaluating the risk of short-term heart failure progression under tafamidis treatment, baseline T1 mapping parameters, rather than their changes, may prove more informative. Although the dataset is limited in size and the follow-up period is short, our findings from the follow-up CMR studies are expected to contribute novel insights into the management of ATTR-CM.

## 5 Limitations

Our study has several limitations. The cohort of patients was limited in size, potentially introducing selection bias. Only 12 patients experienced WHF, which included hospitalization and outpatient diuretic intensification, during the one-year period. This low incidence rate may lead to an overestimation of effect sizes, such as the hazard ratio of T1_native_. Factors such as the presence of advanced chronic kidney disease, history of allergy to gadolinium-based contrast agents, and patient refusal to undergo contrast administration led to a reduction in the number of patients completing both pre- and post-contrast scans. About one-quarter of the study participants did not undergo post-contrast CMR, which may have resulted in non-significant ECV findings. While T1_native_ imaging can be conducted without gadolinium contrast, the determination of ECV requires post-contrast imaging, thereby limiting its utility as a principal imaging marker in the management of ATTR-CM. On the one hand, it must be noted that each institution needs to establish a reference value for normal T1_native_. Furthermore, the optimal cutoff values for T1_native_ and ECV, as identified in this study for predicting WHF, should be validated in an external cohort to assess their clinical applicability. In this study, we retrospectively enrolled patients who received tafamidis treatment and underwent CMR scans. Consequently, there was no control group of patients who did not receive tafamidis. We acknowledge the necessity of comparing an untreated control group with a treated group to further elucidate the effects of treatment on the T1 mapping parameters. However, we adhered to the guidelines for the administration of tafamidis as outlined for patients with ATTR-CM in Japan [2], which made the formation of a control group challenging. Another limitation is the absence of histological data from the myocardium in this study, precluding the examination of the relationships between T1 mapping parameters and histological evidence of amyloid deposition and myocardial degeneration. Finally, extended follow-up is necessary to further elucidate the clinical utility of CMR, particularly concerning the role of changes in T1 mapping parameters, in patients with ATTR-CM undergoing tafamidis treatment.

## 6 Conclusion

In patients with wild-type ATTR-CM undergoing tafamidis treatment, elevated myocardial T1 mapping parameters prior to the treatment were associated with one-year WHF, and T1_native_ emerged as a potential independent predictor of one-year WHF. T1 mapping parameters, particularly T1_native_, may offer imaging-based evidence of alterations in myocardial characteristics induced by tafamidis. Given the absence of correlations between changes in myocardial T1 mapping parameters and one-year WHF, their baseline values, rather than their changes, may have short-term prognostic value in this population. Given the limited sample size, the findings derived from this study should be interpreted with caution, and the clinical utility of myocardial T1 mapping parameters in patients with ATTR-CM undergoing tafamidis treatment necessitates an extended investigation involving a larger cohort.

## 7 Ethics approval and consent

The study protocol was approved by the Ethical Committee for Epidemiology of Hiroshima University (reference number E2018-1364). Data collection was performed retrospectively using medical records. Informed consent was obtained using the opt-out method.

## 8 Availability of data and materials

The datasets used and analyzed during the current study are available from the corresponding author upon reasonable request.

## CRediT authorship contribution statement

**Toshiro Kitagawa:** Writing – original draft, Supervision, Resources, Methodology, Funding acquisition, Formal analysis, Conceptualization. **Kotaro Hamamoto:** Visualization, Validation, Investigation. **Daiki Okamoto:** Visualization, Validation, Investigation. **Yuki Ikegami:** Visualization, Validation, Investigation. **Yoshiharu Sada:** Validation, Supervision, Data curation. **Fuminari Tatsugami:** Resources, Methodology, Data curation. **Yukiko Nakano:** Writing – review & editing, Supervision, Conceptualization.

## Funding

This study was supported in part by a JSPS KAKENHI Grant-in-Aid for Scientific Research (grant number 21K08127).

## Declaration of competing interest

The authors declare that they have no known competing financial interests or personal relationships that could have appeared to influence the work reported in this paper.

## References

[1] Maurer M.S., Schwartz J.H., Gundapaneni B., Elliott P.M., Merlini G., Waddington-Cruz M. (2018). Tafamidis treatment for patients with transthyretin amyloid cardiomyopathy. N. Engl. J. Med..

[2] Endo J., Sano M., Izumiya Y., Tsujita K., Nakamura K., Tahara N. (2019). A Statement on the appropriate administration of tafamidis in patients with transthyretin cardiac amyloidosis. Circ J.

[3] Kitaoka H., Izumi C., Izumiya Y., Inomata T., Ueda M., Kubo T. (2020). JCS 2020 guideline on diagnosis and treatment of cardiac amyloidosis. Circ J.

[4] Fontana M., Banypersad S.M., Treibel T.A., Maestrini V., Sado D.M., White S.K. (2014). Native T1 mapping in transthyretin amyloidosis. JACC Cardiovasc. Imaging.

[5] Karamitsos T.D., Piechnik S.K., Banypersad S.M., Fontana M., Ntusi N.B., Ferreira V.M. (2013). Noncontrast T1 mapping for the diagnosis of cardiac amyloidosis. JACC Cardiovasc. Imaging.

[6] Pan J.A., Kerwin M.J., Salerno M. (2020). Native T1 mapping, extracellular volume mapping, and late gadolinium enhancement in cardiac amyloidosis: a meta-analysis. JACC Cardiovasc. Imaging.

[7] Rettl R., Mann C., Duca F., Dachs T.M., Binder C., Ligios L.C. (2022). Tafamidis treatment delays structural and functional changes of the left ventricle in patients with transthyretin amyloid cardiomyopathy. Eur. Heart J. Cardiovasc. Imaging.

[8] Chamling B., Bietenbeck M., Korthals D., Drakos S., Vehof V., Stalling P. (2023). Therapeutic value of tafamidis in patients with wild-type transthyretin amyloidosis (ATTRwt) with cardiomyopathy based on cardiovascular magnetic resonance (CMR) imaging. Clin. Res. Cardiol..

[9] Ikegami Y., Kitagawa T., Sada Y., Okamoto D., Hamamoto K., Tatsugami F. (2025). Myocardial T1 mapping, left ventricular parameters, and cardiac biomarkers in transthyretin amyloid cardiomyopathy before and after tafamidis treatment. Circ. Rep..

[10] Grogan M., Scott C.G., Kyle R.A., Zeldenrust S.R., Gertz M.A., Lin G. (2016). Natural history of wild-type transthyretin cardiac amyloidosis and risk stratification using a novel staging system. J. Am. Coll. Cardiol..

[11] Gillmore J.D., Damy T., Fontana M., Hutchinson M., Lachmann H.J., Martinez-Naharro A. (2018). A new staging system for cardiac transthyretin amyloidosis. Eur. Heart J..

[12] Lockwood P.A., Le V.H., O'Gorman M.T., Patterson T.A., Sultan M.B., Tankisheva E. (2020). The bioequivalence of tafamidis 61-mg free acid capsules and tafamidis meglumine 4 × 20-mg capsules in healthy volunteers. Clin. Pharmacol. Drug Dev..

[13] Kitagawa T., Tatsugami F., Yokomachi K., Akiyama Y., Fujii Y., Awai K. (2022). Native myocardial T1 value in predicting 1-year outcomes in patients with nonischemic dilated cardiomyopathy experiencing recent heart failure. Int. Heart J..

[14] Garcia-Pavia P., Bengel F., Brito D., Damy T., Duca F., Dorbala S. (2021). Expert consensus on the monitoring of transthyretin amyloid cardiomyopathy. Eur. J. Heart Fail..

[15] Martinez-Naharro A., Kotecha T., Norrington K., Boldrini M., Rezk T., Quarta C. (2019). Native T1 and extracellular volume in transthyretin amyloidosis. JACC Cardiovasc. Imaging.

[16] Duca F., Rettl R., Kronberger C., Binder C., Mann C., Dusik F. (2023). Myocardial structural and functional changes in cardiac amyloidosis: insights from a prospective observational patient registry. Eur. Heart J. Cardiovasc. Imaging.

[17] Nies RJ, Ney S, Nies JF, Seuthe K, Klösges L, Brüwer M, et al. Outpatient diuretic intensification: a simple prognostic marker in cardiac transthyretin amyloidosis. Clin. Res. Cardiol. (2025) in press. 10.1007/s00392-025-02617-4 PMC13083326 40035810

[18] Fontana M., Maurer M.S., Gillmore J.D., Bender S., Aldinc E., Eraly S.A. (2025). Outpatient worsening heart failure in patients with transthyretin amyloidosis with cardiomyopathy in the HELIOS-B Trial. J. Am. Coll. Cardiol..

[19] Misumi Y., Ando Y., Gonçalves N.P., Saraiva M.J. (2013). Fibroblasts endocytose and degrade transthyretin aggregates in transthyretin-related amyloidosis. Lab. Invest..

[20] Michalon A., Hagenbuch A., Huy C., Varela E., Combaluzier B., Damy T. (2021). A human antibody selective for transthyretin amyloid removes cardiac amyloid through phagocytic immune cells. Nat. Commun..

[21] Fontana M., Martinez-Naharro A., Chacko L., Rowczenio D., Gilbertson J.A., Whelan C.J. (2021). Reduction in CMR derived extracellular volume with patisiran indicates cardiac amyloid regression. JACC Cardiovasc. Imaging.

