# Supplementary material for: One-year worsening heart failure and myocardial T1 mapping in patients with wild-type transthyretin amyloid cardiomyopathy undergoing tafamidis treatment
Source: Int J Cardiol Heart Vasc. 2026 Apr 24;64:101934. doi: 10.1016/j.ijcha.2026.101934 (PMC13127272; doi:10.1016/j.ijcha.2026.101934)
Supplement: Supplementary Data 1 [file mmc1.pdf]

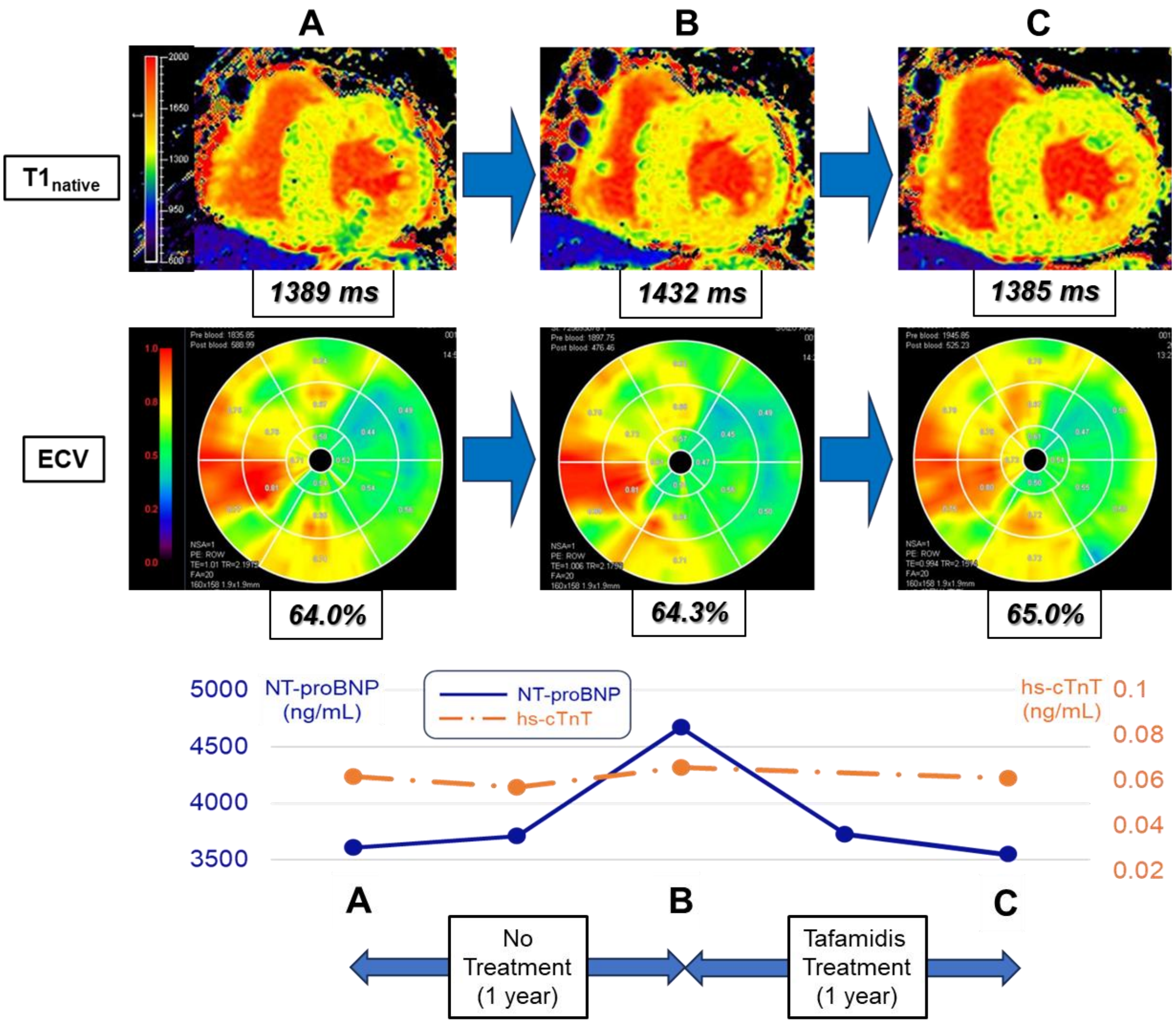

Fig. S1. A representative ATTR-CM patient with data from serial CMR scans and cardiac biomarker measurements before and after tafamidis treatment.

This patient, a 72-year-old male, underwent serial CMR scans and cardiac biomarker measurements one year prior to, at the initiation of, and one year after tafamidis treatment. $T1_{native}$ increased during one-year period before the treatment and decreased after one year of the treatment. On the one hand, ECV was nearly constant during the period before and after the treatment. In parallel with the alteration in $T1_{native}$, the concentration of serum NT-proBNP

increased before treatment and decreased after treatment, whereas the concentration of serum hs-cTnT remained relatively stable.

ATTR-CM − transthyretin amyloid cardiomyopathy; CMR − cardiac magnetic resonance; ECV − extracellular volume fraction; hs-cTnT, high-sensitivity cardiac troponin T**;** NT-proBNP − N-terminal pro-B-type natriuretic peptide; $T1_{native}$ − native myocardial T1 value; WHF − worsening heart failure.
