# Supplementary material for: One-year worsening heart failure and myocardial T1 mapping in patients with wild-type transthyretin amyloid cardiomyopathy undergoing tafamidis treatment
Source: Int J Cardiol Heart Vasc. 2026 Apr 24;64:101934. doi: 10.1016/j.ijcha.2026.101934 (PMC13127272; doi:10.1016/j.ijcha.2026.101934)
Supplement: Supplementary Data 2 [file mmc2.pdf]

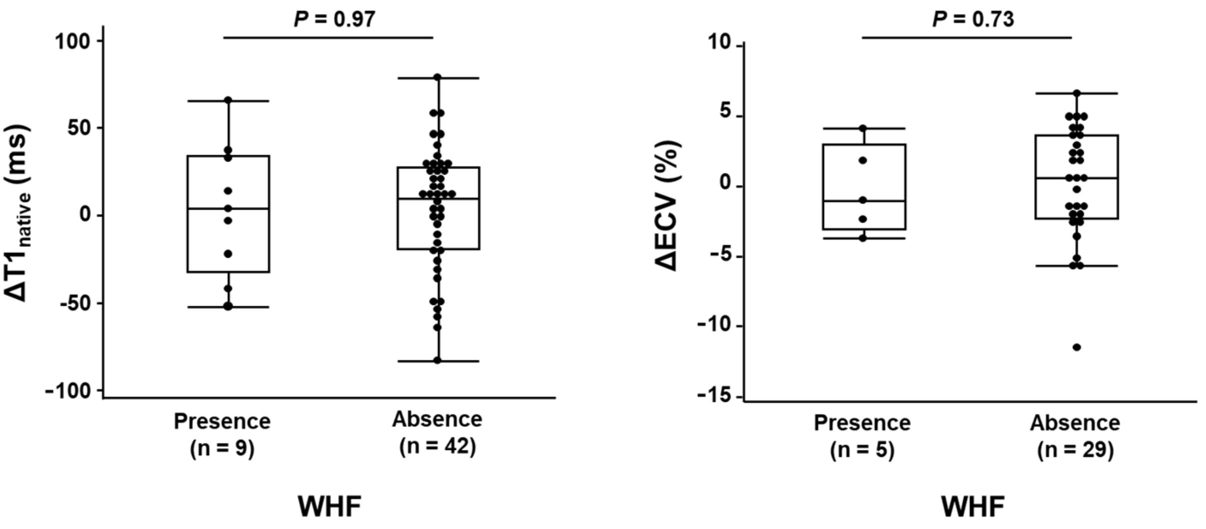

Fig. S2. Comparison of changes in T1 mapping parameters after tafamidis treatment between patients with WHF and those without.

No significant differences were observed between the groups in terms of changes in the $T1_{native}$ or ECV.

ECV − extracellular volume fraction; $T1_{native}$ − native myocardial T1 value; WHF − worsening heart failure.
