# Supplementary material for: One-year worsening heart failure and myocardial T1 mapping in patients with wild-type transthyretin amyloid cardiomyopathy undergoing tafamidis treatment
Source: Int J Cardiol Heart Vasc. 2026 Apr 24;64:101934. doi: 10.1016/j.ijcha.2026.101934 (PMC13127272; doi:10.1016/j.ijcha.2026.101934)
Supplement: Supplementary Data 3 [file mmc3.pdf]

Table S1. Baseline characteristics of the entire cohort and the CMR follow-up group

	Entire		CMR follow-up	
	All patients (n = 60)	Patients with ECV data (n = 46)	All patients (n = 51)	Patients with ECV data (n = 34)
Age (years)	78 ± 5	77 ± 5	78 ± 5	77 ± 5
Male sex	53 (88)	41 (89)	45 (88)	31 (91)
BMI (kg/m^2)	23 ± 3	23 ± 3	23 ± 3	23 ± 3
Heart failure hospitalization within one month	18 (30)	13 (28)	15 (29)	11 (32)
Hypertension	16 (27)	13 (28)	14 (27)	10 (29)
Dyslipidemia	13 (22)	9 (20)	11 (22)	6 (18)
Diabetes mellitus	23 (38)	15 (33)	21 (41)	11 (32)
Current smoking	8 (13)	6 (13)	6 (12)	4 (12)
History of atrial fibrillation	19 (32)	12 (26)	17 (33)	9 (26)

Medications				
Beta-blocker	22 (37)	15 (33)	21 (41)	12 (35)
ACE inhibitor or ARB	26 (43)	21 (46)	25 (49)	17 (50)
MRA	28 (47)	20 (43)	24 (47)	15 (44)
Diuretics	46 (77)	32 (70)	39 (76)	24 (71)
SGLT2 inhibitor	17 (28)	13 (28)	13 (25)	8 (24)
Blood testing				
Hemoglobin (g/dL)	13.6 ± 1.7	14.0± 1.6	13.6 ± 1.7	14.1 ± 1.6
eGFR (mL/min/1.73 m^2)	50.1 ± 15.8	55.6 ± 11.9	50.6 ± 16.4	56.8 ± 12.0
hs-cTnT (ng/mL)	0.060 (0.046−0.082)	0.055 (0.043−0.066)	0.061 (0.044−0.082)	0.055 (0.042−0.066)
NT-proBNP (pg/mL)	2141 (1363−3330)	1745 (979−3028)	2044 (1174−3176)	1660 (975−3028)
LV parameters				
LVEF (%)	54.4 ± 11.3	55.5 ± 10.7	54.8 ± 11.5	54.8 ± 10.8
LVEDVI (ml/m^2)	76.2 ± 16.8	76.2 ± 17.2	75.5 ± 17.3	75.4 ± 17.7

LVMI (g/m^2)	93.3 ± 24.0	92.2 ± 23.3	92.7 ± 23.4	93.1 ± 24.0
$T1_{native}$ (ms)	1424 ± 54	1415 ± 53	1425 ± 54	1419 ± 53
ECV (%)	N/A	50.4 ± 8.4	N/A	51.2 ± 8.7

Serum hs-cTnT and NT-proBNP concentrations are expressed as medians with interquartile range. Other data are expressed as means ± standard deviation or numbers with percentage. ACE, angiotensin converting enzyme;ARB, angiotensin II receptor blocker; BMI, body mass index; CMR, cardiac magnetic resonance; ECV, extracellular volume fraction; eGFR, estimated glomerular filtration rate; hs-cTnT, high-sensitivity cardiac troponin T; LV, left ventricular; LVEDVI, LV end-diastolic volume index; LVEF, LV ejection fraction; LVMI, LV mass index; MRA, mineralocorticoid receptor antagonist; NT-proBNP, N-terminal pro-brain natriuretic peptide; SGLT2, sodium glucose cotransporter 2; $T1_{native}$, native myocardial T1 value.
