# Supplementary material for: One-year worsening heart failure and myocardial T1 mapping in patients with wild-type transthyretin amyloid cardiomyopathy undergoing tafamidis treatment
Source: Int J Cardiol Heart Vasc. 2026 Apr 24;64:101934. doi: 10.1016/j.ijcha.2026.101934 (PMC13127272; doi:10.1016/j.ijcha.2026.101934)
Supplement: Supplementary Data 4 [file mmc4.pdf]

Table S2. Baseline characteristics of the entire cohort categorized based on the optimal cutoff values of T1 mapping parameters in predicting WHF

($T1_{native}$)

	$T1_{native} \geq 1447$ ms (n = 20)	$T1_{native} < 1447$ ms (n = 40)	p value
Age (years)	78 ± 4	78 ± 5	0.62
Male sex	18 (90)	35 (88)	0.78
BMI (kg/m^2)	23 ± 3	23 ± 3	0.74
Heart failure hospitalization within one month	5 (25)	13 (33)	0.55
Hypertension	5 (25)	11 (28)	0.84
Dyslipidemia	4 (20)	9 (23)	0.82
Diabetes mellitus	7 (35)	16 (40)	0.71
Current smoking	1 (5)	7 (18)	0.18
History of atrial fibrillation	9 (45)	10 (25)	0.12

Medications			
Beta-blocker	6 (30)	16 (40)	0.45
ACE inhibitor or ARB	12 (60)	14(35)	0.065
MRA	11 (55)	17 (43)	0.36
Diuretics	14 (70)	32 (80)	0.39
SGLT2 inhibitor	5 (25)	12 (30)	0.69
Blood testing			
Hemoglobin (g/dL)	13.0 ± 1.2	14.9 ± 1.8	0.044
eGFR (mL/min/1.73 m^2)	50.3 ± 17.5	50.0 ± 15.2	0.95
hs-cTnT (ng/mL)	0.067 (0.052−0.11)	0.056 (0.044−0.079)	0.078
NT-proBNP (pg/mL)	2140 (1490−3582)	2198 (978−3184)	0.65

Mayo stages			0.42
I	4 (20)	14(35)	
II	10 (50)	14 (35)	
III	6 (30)	12 (30)	
NAC stages			0.27
I	10 (50)	21 (53)	
II	5 (25)	15 (37)	
III	5 (25)	4 (10)	
LV parameters			
LVEF (%)	50.4 ± 11.2	56.4 ± 11.0	0.053
LVEDVI (ml/m^2)	86.1 ± 12.8	71.2 ± 16.5	0.0008
LVMI (g/m^2)	106.3 ± 27.5	86.8 ± 19.4	0.0023

(ECV)

	ECV ≥ 48.7 % (n = 22)	ECV < 48.7 % (n = 24)	p value
Age (years)	76 ± 5	78 ± 5	0.27

Male sex	21 (95)	20 (83)	0.19
BMI (kg/m^2)	22 ± 2	23 ± 3	0.27
Heart failure hospitalization within one month	6 (27)	7 (29)	0.89
Hypertension	6 (27)	7 (29)	0.89
Dyslipidemia	2 (9)	7 (29)	0.086
Diabetes mellitus	6 (27)	9 (38)	0.46
Current smoking	1 (5)	5 (21)	0.10
History of atrial fibrillation	7 (32)	5 (21)	0.40
Medications			
Beta-blocker	3 (14)	12 (50)	0.0086
ACE inhibitor or ARB	11 (50)	10 (42)	0.57
MRA	12 (55)	8 (33)	0.15
Diuretics	17 (73)	15 (63)	0.28
SGLT2 inhibitor	7 (32)	6 (25)	0.61

Blood testing			
Hemoglobin (g/dL)	13.6 ± 1.7	14.3 ± 1.5	0.13
eGFR (mL/min/1.73 m^2)	54.8 ± 11.1	56.4 ± 12.7	0.66
hs-cTnT (ng/mL)	0.059 (0.051−0.079)	0.050 (0.033−0.064)	0.025
NT-proBNP (pg/mL)	2141 (1483−3381)	1435 (878−2829)	0.12
Mayo stages			0.15
I	5 (23)	12 (50)	
II	10 (45)	8 (33)	
III	7 (32)	4 (17)	
NAC stages			0.50
I	12 (55)	17 (71)	
II	9 (41)	6 (25)	
III	1 (5)	1 (4)	
LV parameters			

LVEF (%)	53.9 ± 10.3	57.0 ± 11.1	0.32
LVEDVI (ml/m^2)	81.1 ± 14.4	71.8 ± 18.6	0.067
LVMI (g/m^2)	100.9 ± 25.6	84.2 ± 18.1	0.014

Serum hs-cTnT and NT-proBNP concentrations are expressed as medians with interquartile range. Other data are expressed as means ± standard deviation or numbers with percentage. ACE, angiotensin converting enzyme; ARB, angiotensin II receptor blocker; BMI, body mass index; ECV, extracellular volume fraction; eGFR, estimated glomerular filtration rate; hs-cTnT, high-sensitivity cardiac troponin T; LV, left ventricular; LVEDVI, LV end-diastolic volume index; LVEF, LV ejection fraction; LVMI, LV mass index; MRA, mineralocorticoid receptor antagonist; NAC, the National Amyloidosis Center; NT-proBNP, N-terminal pro-brain natriuretic peptide; SGLT2, sodium glucose cotransporter 2; $T1_{native}$, native myocardial T1 value; WHF, worsening heart failure.
